# Virtual social interaction reveals that the dorsal habenula-IPN pathway is essential for targeting the opponent

**DOI:** 10.1016/j.isci.2026.115566

**Published:** 2026-04-02

**Authors:** Tanvir Islam, Makio Torigoe, Yuki Tanimoto, Ming-Yi Chou, Hitoshi Okamoto

**Affiliations:** 1 RIKEN Center for Brain Science, 2-1 Hirosawa, Wako-shi, Saitama 351-0198, Japan; 2 School of Advanced Science and Engineering, Waseda University, 2-2 Wakamatsu-cho, Shinjuku-ku, Tokyo 169-8555, Japan; 3 Department of Life Science, National Taiwan University, Taipei, Taiwan; 4 Institute of Neuropsychiatry, 91 Benten-cho, Shinjuku-ku, Tokyo 162-0851, Japan

**Keywords:** health sciences, neuroscience, social sciences

## Abstract

Conspecific social interaction is among the most important brain functions to be investigated. We designed a virtual reality (VR) system that enables real-time teleportation of head-fixed adult zebrafish movements onto their 3D avatars. To facilitate dyadic fight-like behavior in the VR space, we devised a task in which avoidance of periodic mild electric stimulation is achieved by attacking the opponent, and a transient electric shock acts as negative feedback for bite-like events. Moreover, a transgenic fish with an impaired dorsolateral habenula-to-interpeduncular nucleus pathway, known as a winner’s circuit in social conflict, showed reduced ability in placing the opponent in the field of binocular view and attacking the opponent, while also showing increased use of the left eye. These indicate the importance of the winner’s circuit in correctly aligning the body axis for capturing the opponent. The proposed VR system can be effective in investigating how the brain encodes various social interactions.

## Highlights

• A VR system for adult zebrafish is designed to investigate dyadic fish-fight
• A novel method of electrical stimulation enhances antagonistic fish interaction
• Transgenic fish with silenced dHbL-d/iIPN pathway show reduced use of binocular vision
• The dHbL-d/iIPN pathway is important for forming binocular vision and target-directed posture

## Graphical abstract

**Figure:**
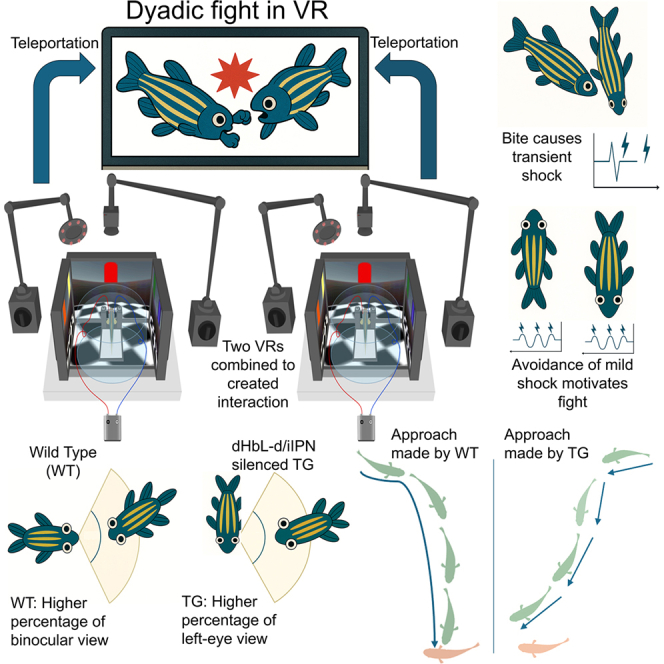

## Introduction

Social interactions are crucial for the survival of animals as they enhance protection from predators, facilitate cooperative group tasks such as hunting and resource sharing, contribute to raising offspring, and thus ultimately increase reproductive success and overall fitness. Even in humans, long-term habits or day-to-day actions contain elements of social behavior that define many aspects of our individual lives and society. As many of these social behaviors require the correct identification and appropriate engagement with conspecifics as well as avoidance of predators, the neural coding of such recognition and action across species such as flies, mice, and primates^1^^,^^2^^,^^3^ has been studied with great interest. Attraction toward conspecifics results in collective behavior such as shoaling in many fish species, including zebrafish, which adopt such behavior typically by 14–21 days post-fertilization (dpf).^4^ While previous research attempted to quantify social attraction through laboratory experiments where multiple live fish separated from the subject by a transparent barrier have been used as stimuli, more recent works tried the integration of engineered, artificial stimuli in the form of still images, videos, and computer-animated 3D images.^5^^,^^6^^,^^7^ In these works, zebrafish seemed to prefer the naturalistic motion of conspecific videos over still images and random movements of dots,^5^ even though video playback did not always create a clear preference over a live shoal.^6^ Furthermore, being chased by a 3D animated image^7^ generated preference for chase-dependent motion in zebrafish. Use of artificial images as cues shows that zebrafish consider various visual features of the stimulus, namely the size, color, shape, and motion, to grow attraction toward the stimulus. These works indicate that a combination of realistic stimuli and locomotion is important to motivate live zebrafish subjects to express appropriate social behavior.

Virtual reality (VR) has been another recent approach in neuroscience to create a more realistic three-dimensional representation of the social environment.^8^^,^^9^ Due to the high flexibility of modifying the color, texture, size, and shape of the surrounding environment, as well as the artificial 3D models of animals that the subject can interact with, VR overcomes several limitations of video playback and computer-animated images. However, to increase the immersiveness of VR and provide motivation to the subject to adapt to the inherent mechanism of movement in the VR, animal model-based stimuli introduced in the VR need to have realistic locomotion; as such, realistic stimuli can invoke the engagement of the subject in active social interaction within the VR. One effective method to create realistic locomotion is to apply “teleportation” of the movement of another living animal to the VR in real time, thus creating a virtual environment in which two subject animals act as visual stimuli for each other and can see each other’s movement in the VR simultaneously. The initial experimental work applying this method showed that larval zebrafish could interact with each other through their teleported representations, which appeared as black dots,^10^ with a subsequent imaging study^11^ revealing the role of a tectothalamic pathway in facilitating the visual recognition of conspecifics. In this work, the motion of other independent subjects residing in separate bowls was instantaneously projected as moving dots on a screen below a transparent bowl in which the larva fish could swim freely, thus enabling multiple subjects to interact with each other. Biologically inspired robots offer a promising alternative to VR by affording the delivery of physical, easily controllable, three-dimensional stimuli.^12^^,^^13^^,^^14^^,^^15^ The feasibility of remote interaction between zebrafish and honeybees in binary decision-making tasks through biologically inspired robots (a zebrafish replica and two bee-robots) has been demonstrated,^16^ in which the authors showed that by controlling the zebrafish replica through spatial density of honeybees and the bee-robot through the swimming direction of zebrafish, it was possible to establish a link between these two species. In a more recent study, a complete mapping of ethograms onto a three-dimensional zebrafish replica was demonstrated^17^ to show that behavioral teleportation can preserve natural interaction between two live animals. The above-mentioned works realize social interaction among freely moving animals. However, to conduct optical imaging to investigate the neural encoding of social interaction, head fixation within immersive stimuli is required. A pioneer VR system for adult zebrafish was proposed^18^ years earlier for head-fixed adult zebrafish. Furthermore, we also previously developed head-fixed VR systems for adult zebrafish that demonstrated the ability of learning a context-dependent go/no-go^19^ task that required the zebrafish to respond to a “go” stimulus while withholding a response to a “no-go” stimulus, and a Morris Water Maze-like task^20^ that required finding a hidden safe-zone in a two-dimensional VR space. To investigate affiliative social interaction in VR, realistic zebrafish models that act as standalone stimulants or as avatars of live zebrafish are required. While several recent studies have used standalone zebrafish images or movies,^6^ as well as moving 3D animations,^7^ a highly realistic 3D model of zebrafish was used very recently as stimulant to induce affiliative responses^21^ in zebrafish. On the contrary, with a focus on the dyadic fighting between male adult zebrafish, we implemented behavioral teleportation by combining two identical VR systems for head-fixed zebrafish. In our system, two zebrafish can simultaneously move around in the VR space and see each other’s avatars in the form of 3D zebrafish models. To make it more realistic, we created these 3D models with textures of original zebrafish skin pigmentation and shape. The complete ethogram of each fish was transported across VRs within a fraction of a second, thereby establishing a novel VR-based paradigm of interaction between two separately located live zebrafish.

**Figure 1:**
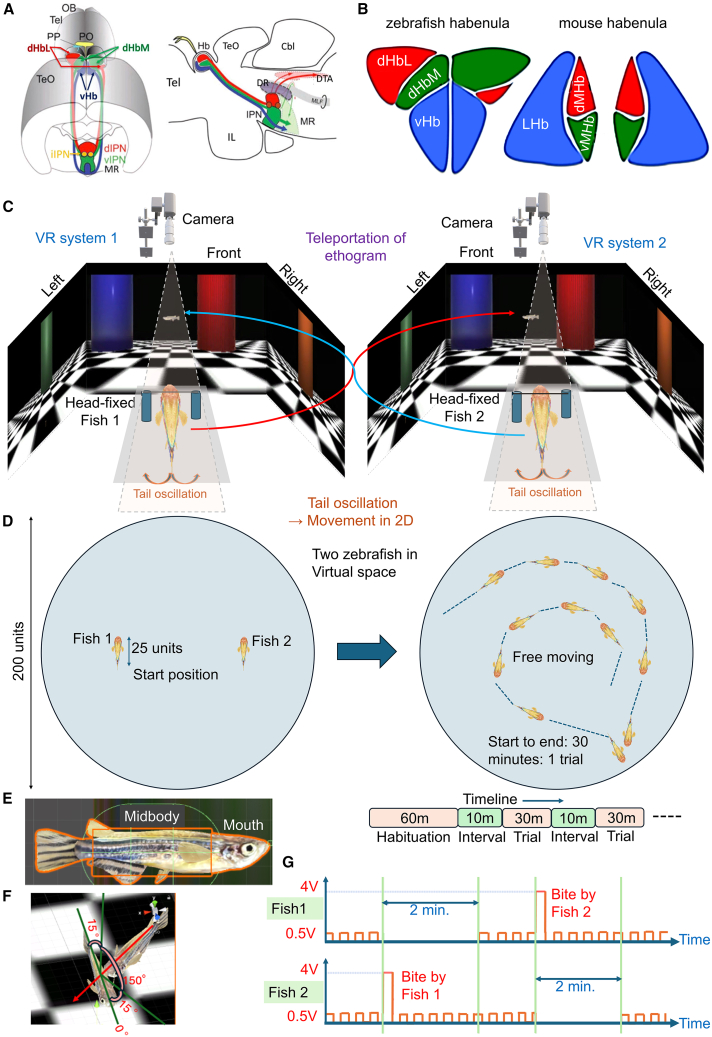
Virtual reality system for interaction between zebrafish and a 3D model of zebrafish and conditions for bite-like behavior (A) Schematic illustration showing dorsal oblique view (left panel) and lateral view (right panel) of the zebrafish dHb-IPN pathways. OB: olfactory bulb; Tel: telencephalon; P: pineal organ; PP: parapineal organ; TeO: tectum opticum; Cbl: cerebellum; IL: inferior lobe of hypothalamus; MR: median raphe; DR: dorsal raphe; DTA: dorsal tegmentum area; MLF: medial longitudinal fascicle. (B) Comparison between the zebrafish and mouse Hb subnuclear structure. The evolutionary corresponding structures are labeled with the same colors both in the zebrafish and mouse habenula. dHbL, the lateral subregion of the dorsal habenula; dHbM, the medial subregion of the dorsal habenula; vHb, the ventral habenula; dMHb, the dorsal subregion of the medial habenula; vMHb, the ventral subregion of the medial habenula; LHb, the lateral habenula. (C) A combination of two VR systems is used for two adult head-fixed zebrafish whose ethograms are transported in real time. Each zebrafish can see the moving avatar of the other zebrafish in the VR and adapt its tail movements for interaction. Forward speed and turning amount are extracted from tail oscillation, which is captured by a camera. (D) Combination of two VR systems with the transportation of the ethogram facilitates both zebrafish to experience being together in a circular virtual space of diameter = 200 units. The start position of the two fish at the start of a trial (left diagram) is a sidewise position separated by 100 units, with the locations (50,0) and (−50,0) in the two-dimensional axis system. After the start of a trial, both fish can move around freely while observing each other with a first-person view and interacting with each other (right diagram). Timeline of the experiment is shown below. (E) 3D model of zebrafish created with textures taken from images of real zebrafish. A “mid-body” section of the avatar is defined in the middle part of the body, and the tip of the head is defined as the “mouth.” The length of the 3d zebrafish model is 25units. (F) Conditions for a collision to be considered as a bite are depicted. A collision with the mouth with the mid-body of the avatar of the other fish with an angle of inclination of ±75^o^ with respect to the body axis is considered as a bite. (G) Mechanism of the electric shock that was administered in the shock trials. Mild periodic electric shock of 0.5V is introduced at the beginning of the trial. The duration of the mild shock is 300 ms per second. If one fish bit the other, then the “bitten” fish would receive 4V of transient shock followed by the continuation of the mild electric shock, and the fish that performed the bite is relieved of shock for 2 min.

Among the various forms of social interaction that our VR system can facilitate, it has been of our particular interest to investigate the neural coding of dyadic fish fight in adult male zebrafish. Dyadic fights, or antagonistic interactions between two conspecific individuals, are a common form of social behavior, often seen in play fighting, aggression, and competition, and can be studied to understand social dynamics and the development of social skills. Zebrafish exhibit dyadic fighting behavior,^22^ a stereotyped sequence of interactions used to establish dominance, where winners often display active behaviors such as chasing and biting, while losers show passive behaviors such as fleeing. Zebrafish habenula (Hb) plays a major role in social fighting.^23^^,^^24^ In zebrafish, the Hb can be divided into two subnuclei: the dorsal habenula (dHb) and ventral habenula (vHb) (Figure 1A). The dHb is considered the homolog of the mammalian medial habenula (MHb). The lateral subregion of the dHb (dHbL) in zebrafish, (Figure 1B) projects to the dorsal and intermediate IPN (d/iIPN), whose axons extend through several regions of the dorsal tegmental area (DTA)^25^ that contains the area corresponding to the mammalian periaqueductal gray (PAG). In mammals, the circuitry involving IPN, the PAG, and the nucleus incertus has been implicated in the regulation of behaviors such as aversion^26^ and pain, defensive and aggressive behaviors, anxiety, and depression.^27^ Recent studies have been further revealing the role of Hb for controlling behaviors in social conflict. For example, it has been shown recently^28^ that the neural pathway from the superior part of the medial Hb (MHbS) to the lateral subnucleus of the IPN (LIPN) is activated under anxiogenic environments to counteract anxiety in mice. Another very recent study on mice^29^ elucidates how the Hb-IPN-median raphe pathway regulates the outcome of social dominance conflicts. In the case of zebrafish, previous work^23^ shows that the potentiation of the dHbL-d/iIPN-DTA pathway facilitates the winner behavior, and a reduction in the transmission of this pathway with an accompanying reduction in the activity propagation to the DTA contributes to giving up continuation of attack and promotes surrender. The dHbL-d/iIPN-DTA pathway (Figure 1A) also contributes to the modulation of fear behaviors.^25^ The transgenic mutant zebrafish used in these studies is a laboratory-made dHbL-silenced transgenic line *Tg(narp:GAL4VP16); Tg(UAS: tetanus neurotoxin light chain (TeTxLC))*, in which the neurotransmission in the dHbL-d/iIPN pathway was selectively inhibited by Tetanus toxin expression. Further work with this transgenic fish (to be denoted as TG fish from here onwards) revealed a specific defect in learning cognitive tasks that require proper processing of the internal-directional information, i.e., distinguishing one’s left and right, while preserving the ability to learn tasks where correct recognition of external cues (blue or red color of the goals) is associated with task rules.^30^ The VR system we propose here will also enable simultaneous optical imaging of the neural activities of two adult zebrafish while they interact with each other. We initially used head-fixed wild-type (WT) fish pairs (WT-WT pairs) to investigate how two zebrafish interact with each other in VR. As adult zebrafish males engage more vigorously in fish fights than females, we used only adult male zebrafish as subjects of our study. Moreover, to increase the motivation for fighting, we provided periodic mild electric stimuli that could be avoided only by approaching closely enough to hit the opponent in a simulated biting behavior. Furthermore, during the biting-like behavior, a transient electric shock was provided to the opponent as feedback for being “bitten.” To investigate the behavior patterns of the TG fish in dyadic fights, we used TG-WT fish pairs and investigated the differences between the TG and WT fish during fighting-like social interaction. Moreover, we investigated and analyzed the laterality^31^^,^^32^ in zebrafish visual information processing during approaches toward the target.

## Results

### A VR system for using avatars of multiple fish is constructed to investigate social interaction

To investigate the interaction between two head-fixed adult zebrafish, we have established a VR system (Figure 1C) that can be used in combination with optical imaging microscopes. This system consists of two separate VR sub-systems, each of which is used for one head-fixed zebrafish.^20^ The programmatic design of the system (see STAR Methods) allows individual zebrafish to roam around in a circular VR arena either alone or in pairs, with the 3D avatar of the paired fish visible inside the VR. For initial adaptation to the head-fixed condition in the VR, each fish swam alone for 1 h. After that, two fish moved around in the virtual space together in trials, each being 30 min long. During the trials, the movement of each fish, which consists of forward speed and turning, was teleported to the VR screens of the other paired fish. This resulted in a VR space in the form of a circular arena of a radius (Figure 1D) of 100 units. At the start of the trial, the two zebrafish were aligned 100 units apart and positioned in parallel to each other, at locations (50,0) and (−50,0) in the XY coordinate system of the VR space (Figure 1D, left). This allowed zebrafish to approach each other and interact (Figure 1D, right). The 3D models and the surrounding wall of the VR arena were non-penetrable objects that did not allow other objects to pass through, therefore causing collisions with physics-simulated properties allowed by the Unity 3D game engine. The length of the fish avatar was 25 units, which is roughly one-eighth of the diameter of the VR arena.

### Mechanisms to stimulate dyadic fight-like behavior are introduced

Previously, it was found that zebrafish perform dyadic fights in real-world interaction.^22^^,^^23^ This kind of fight requires fast, circular movement around each other with attempts at biting. While such fast circular motion involving the bending of head and tail is difficult in the head-fixed condition, we still expected that the 3D avatars (Figure 1E) of the opponent fish could invoke elements of dyadic fight, for example, approaching and biting behavior. To define such behavior in VR, we assigned a collision property to the middle part of the body and the mouth sections (named as “mid body” and “mouth” in Figure 1E) of the avatar. In our system, each fish observed the avatar of the opponent with a first-person view. If the “mouth” section of one fish collided with the “midbody” section of the opponent in such a way that the angle of inclination with respect to the line perpendicular to the body axis of the opponent fish falls within the range of ±75° (Figure 1F), the collision was considered a bite. A bite was counted each time such a collision occurred, given that the previous frame did not generate a collision. In this way, collisions lasting over subsequent frames were counted as only one bite.

To induce motivation for biting and to provide feedback, we introduced a rule based on electric shock (Figure 1G). To motivate biting, both fish were given a low-voltage periodic electric stimulus (0.5V) from the beginning of each trial. The periodic stimulus was on for 300 ms every second. At the event of a bite, the fish that was “bitten” received an instantaneous shock of 4V for 100 ms, followed by the periodic low-voltage electric stimuli. On the other hand, the fish that performed the bite was relieved from the low-voltage periodic stimuli for 2 min, after which the low-voltage stimuli resumed. To compare the effect of this scheme, we used two types of trials for all pairs of fish. The initial few trials were “no-shock” trials, where neither the low voltage stimuli nor the instantaneous shock was administered throughout the trials. These no-shock trials were succeeded by the “shock” trials, where fish were subjected to both types of electric shock.

### Approaches made by individual fish are defined and extracted

**Figure 2:**
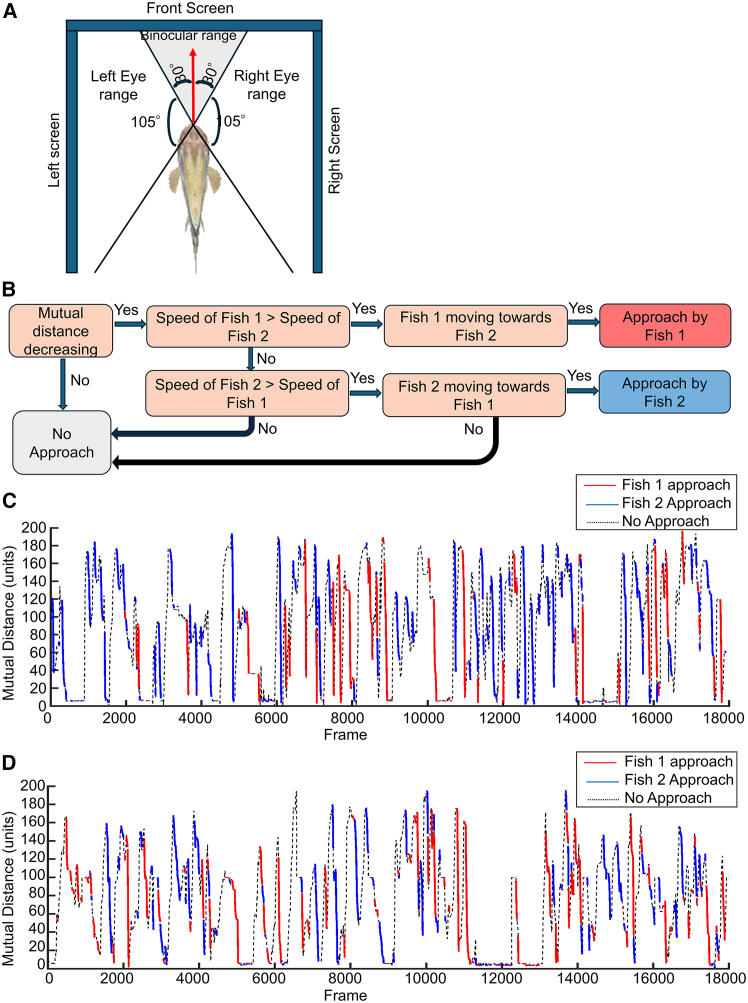
Definition of the left eye view, right eye view, and binocular view, as well as the detection of approaches in VR (A) Schematic of the VR setup. The three screens (left, front, right) show the views of three virtual VR cameras, each with a horizontal view range of 90 °, thus comprising a 270 ° view. The left eye and the right eye view ranges are defined as ranges of −135^o^ ∼ -30^o^ and 30^o^∼135^o^, respectively, whereas the binocular view was defined with a viewing range of −30^o^∼30^o^. For example, if the avatar of the paired fish was within the −30^o^∼30^o^ range of vision, it was supposed that the paired fish was viewed with binocular view. (B) Logic flowchart shows the definition of approach episodes. An approach episode was assigned to fish1 if (1) the mutual distance between fish1 and fish2 kept reducing over the episode, (2) Fish1 had a faster speed than fish2, and (3) Fish1 moved toward fish2. (C and D) show examples of detection of approaches that are assigned to individual fish, with different colors superimposed on the mutual distance graph.

To investigate how fish view each other in VR, we first defined the range of the left and the right eye view, as well as the range in which binocular vision is possible. Instead of tracking the eye movements of fish, we divided the total horizontal viewing range (total 270^o^, comprising of the left, front, and right cameras) of the VR scene as follows: left eye view ranges from −135^o^ ∼ -30^o^, field of binocular view, ranging from −30^o^∼30^o^, and right eye view ranges from 30^o^ to 135^o^ (Figure 2A). The field of Binocular view (FBV), is chosen as ±30^o^ based on the increased probability of binocular view use in larval zebrafish during pray capture,^33^ due to eye convergence. If the opponent fish was within an angular range of −135^o^ ∼ -30^o^ in one specific frame, that frame was considered as one count of left eye view of the opponent. However, in cases when the opponent was outside any of the above-mentioned angular ranges, the opponent was defined to be in the blind spot and not visible.

To extract episodes in which fish were moving toward their opponents and not moving away, we defined three conditions that should be met in an approach (Figure 2B). In this diagram, the two paired fish are denoted as Fish 1 and Fish 2. To define an episode as an approach made by Fish 1, (1) The mutual distance had to be decreasing throughout the episode, (2) The average speed of Fish 1 in the episode was higher than that of Fish 2, and (3) Fish 1 had to be moving toward Fish 2, which meant Fish 2 being in the ±90^o^ viewing range of the approaching Fish 1 throughout the episode. These three conditions for the approach ensured that each approach was assigned to only one fish. It can be noted that when both fish were moving toward each other, the approach was assigned to the fish with the higher speed, and there was no approach where the speed of the two fish was the same. Examples of approaching episodes are illustrated in colors in the graphs of mutual distance in two trials (Figures 2C and 2D), where red and blue sections represent the approaching episodes assigned to the two fish, and dotted lines indicate the parts where no approach was made.

### Higher attraction and lower mutual distance can be observed in shock trials

**Figure 3:**
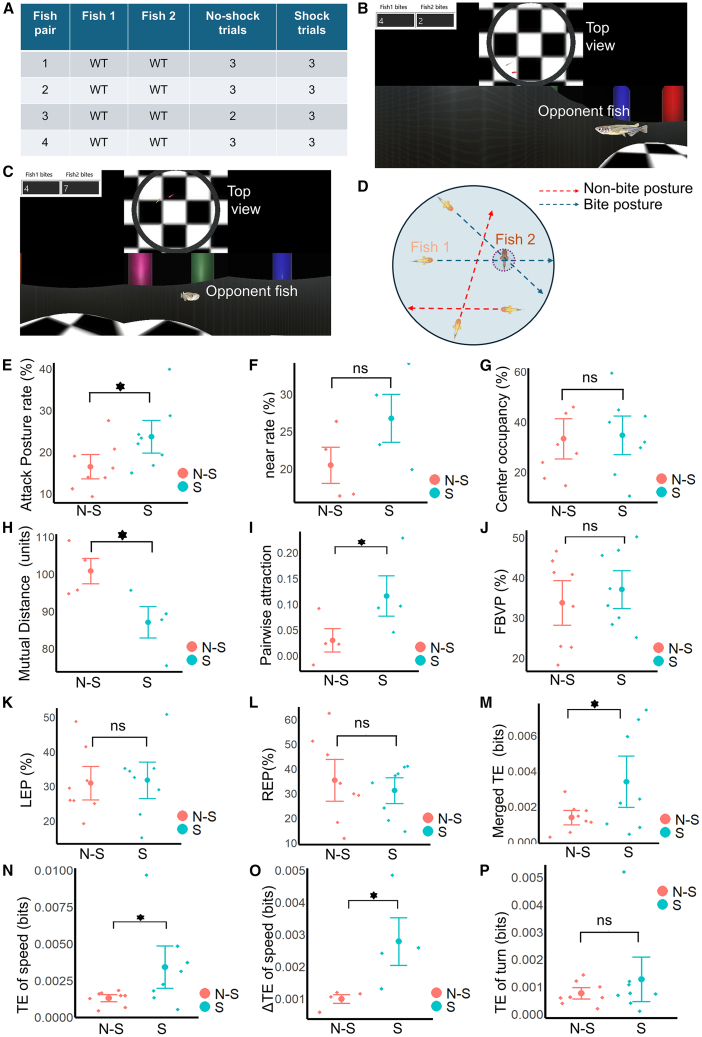
Comparison between no-shock and shock conditions in WT fish While comparing between no-shock and shock conditions, throughout this figure, red and blue denote no-shock and shock, respectively. Enlarged points at the center of the vertical red and the blue lines denote mean values, and the two ends represent error bars (mean ± 0.5∗standard deviation). Individual fish or pairs are denoted as single data points. (A) The number of no-shock and shock trials conducted with four pairs of WT fish. Shock trials were conducted after the no-shock trials finished. For each pair, corresponding trials were performed on the same day. (B) Conceptual diagram depicting the attack posture. If an extension of the present head direction passed through the circle encompassing the avatar of the other fish (blue dashed lines), the present posture was counted as an attack posture. If the direction was not toward the other fish, then the posture was not considered as an attack posture (red dashed lines). (C and D) These are screenshots from Video S1, showing the opponent fish from the viewpoint of the approaching fish (marked in red in the top panel that shows the top view of the VR arena). (E) Comparison of the attack posture rate of WT fish in no-shock and shock conditions. The Attack Posture rate was calculated as the percentage of bite-posture frames in a trial. (F) Near rate of no-shock and shock trials. Near rate was calculated as the percentage of frames in which the mutual distance between two fish was less than 50 units. (G) Center occupancy rate of no-shock and shock trials. Center occupancy rate was calculated as the percentage of frames in which the fish was within the inner circle of the VR arena. (H) Mutual distance between fish in no-shock and shock trials. Mutual distance of one trial was calculated by averaging the mutual distance values of all frames in that specific trial. (I) Attraction between fish in no-shock and shock trials. (J) Comparison of FBVP of approaches in the no-shock and shock conditions. Approach data are pooled from the fish of four WT-WT pairs. (K) Comparison of LEP in no-shock and shock approaches. (L) Comparison of REP in no-shock and shock conditions. (M) Comparison of TE values in no-shock and shock values when increases and decreases in both the speed and the head direction are considered (merged ethogram). (N) TE in no-shock and shock conditions when only speed values are considered. (O) Comparison between ΔTE values in the no-shock and shock conditions, when only speed values are considered. (P) Comparison between TE values in no-shock and shock trials when only the turning angle is considered.

We used four pairs of WT fish that interacted with each other under both no-shock and shock trials, as shown in the table of Figure 3A. Between two consecutive trials, a 10-min break was administered. In all pairs, no-shock trials preceded the shock trials. All trials were conducted during the daytime, between 10:00 a.m. and 5:00 p.m., in a dark experimental room where only the visible lights from the displays used in the VR were present.

In the four WT-WT pairs, fish on average made ∼35 approaches per one no-shock trial and ∼38 approaches per one shock trial. An example of interaction between two zebrafish is shown in Video S1. In this example, two zebrafish can be seen moving in the VR space while approaching each other and exhibiting bite-like behavior. Two screenshots from this video are shown in Figures 3B and 3C, where the opponent fish is seen from the observer fish’s first-person view, marked in red in the upper panel, which shows the top view of the circular VR arena. Another example video of interaction, showing the plots of head directions (HDs) and the trajectories of two WT fish, is presented in Video S2.

Video S1. Example video of interaction between two zebrafish in virtual space In the movie, a top view of the movements of the two zebrafish inside the circular arena is shown from a third-person view in the upper panel, with one fish colored red. The left, front, and right parts of the VR scene are shown in the bottom panel, with the first-person view of the red colored fish. The numbers in the upper left inset are the total number of bites performed by the two fish. The movie is shown at 5× speed.

Video S2. Example trajectories of zebrafish in VR Moving directions and trajectories of two WT fish are shown in the left and the right panels, respectively. One fish is marked white, while the other is marked red. Interaction between two WT fish can be observed. The movie is shown at 3× speed.

In the following sections, we have made rigorous statistical comparisons between the “no-shock” and “shock” conditions within the eight WT fish (Figure 3A), as well as comparisons between the WT and TG fish groups as well as between the WT-WT and WT-TG pairs. The sample numbers used in each statistical comparison are given in Table S1, where it is mentioned whether data-points used for the comparison are single fish (one average value calculated from one fish) or a pair (one average value calculated from one pair).

We formulated the following indices to characterize the behavior of WT zebrafish in the virtual arena and compared their values in the no-shock and the shock trials using the Wilcoxon Signed Rank test. The “Attack posture rate” of a fish in a trial was defined (see STAR Methods) as the percentage of frames in which the HD of that fish in VR space was such that an extension of that direction would go through a small circle surrounding the avatar of the other fish (blue dashed lines, Figure 3D). We found that the attack posture rate was significantly higher in the shock condition (Figure 3E) than in the no-shock condition (*p* = 0.04003, effect size = 0.93907). However, the “‘near rate,” which is the percentage of frames in which the distance between the two fish was less than 50 units (see STAR Methods), was not significantly different (Figure 3F) between no-shock and shock conditions (*p* = 0.10062). Again, the “center occupancy rate,” which is defined as the percentage of frames in which zebrafish was inside the inner circle (an imaginary circle of a radius of 70 units that had half of the area of the circular virtual arena), did not change significantly between no-shock and shock conditions (Figure 3G) of WT-WT pairs (*p* = 0.41682).

Mutual distance of a trial was calculated by measuring the inter-fish distance in each frame and averaging them over the trial length. Even though we did not observe any reduction or speed in no-shock and shock conditions (no statistical difference was observed, Figure S1), significantly lower mutual distance (Figure 3H) between the paired fish was observed in the shock condition (*p* = 0.03030, effect size = 1.34398). We also measured the mutual attraction^10^ (see STAR Methods) between fish, which measures the percentage of the mean mutual distance that decreases with respect to the average of 10 measurements of mutual distance that were calculated from randomly shuffled position data of one of the two fish. We found that mutual attraction was higher in the shock condition (*p* = 0.0317, effect size = 1.14645), as shown in Figure 3I.

We also conducted simulations by the permutation of the positions of one fish at a random time point, 100 times for each trial, and found that the original distance is always lower than the distance values of the permuted data, and the original near rate is always higher than the permuted data. A couple of examples are shown in Figures S2A and S2B, and comparisons within all WT fish trials are shown in Figure S2C.

### Comparison of the field of binocular view percentage (FBVP), the Left Eye Percentage (LEP), and the Right Eye Percentage (REP) between the no-shock and the shock condition

As we have defined the angular ranges of the left and the right eyes, and the field of binocular view previously (shown in Figure 2A), here we create three indices regarding how the opponent is viewed in VR. First, the left eye percentage (LEP) is defined as the percentage of frames in which the opponent fish is viewed within the left eye range. Similarly, the right eye percentage (REP) is defined as the percentage of frames in which the opponent fish is within the right eye range. Unlike LEP and REP, we could not determine whether the opponent, while being placed within the field of binocular view, was viewed with binocular vision for certain. Therefore, we measured the percentage of frames in which the opponent fish was within the range of binocular vision and named it the field of binocular view percentage (FBVP). With these definitions, we compared the FBVP, LEP, and REP in no-shock and shock conditions. Details of these measurements are mentioned in the STAR Methods. FBVP did not change significantly in shock trials (Figure 3J, *p* = 0.21545) compared to no-shock trials. Furthermore, we did not see a statistically significant difference in the LEP of the WT-WT pairs (Figure 3K, *p* = 0.41682) as well as the REP (Figure 3L, *p* = 0.36314) between no-shock and shock conditions.

### Transfer entropy (TE) is increased during shock trials

Transfer Entropy (TE)^34^ (see STAR Methods) is a statistical measure to estimate the amount of information flowing from one process to another, thus calculating the inter-process “influence” or quantifying the causality between two processes (see STAR Methods). In general, TE from a process *X* to a process *Y* is the decrease of uncertainty in predicting future values of *Y* by knowing past values of both *X* and *Y*, compared to predicting future values of *Y* from only the past values of *Y*. Here, we opted to measure the flow of information between the movements of two fish by using transfer entropy. First, we compared the TE of no-shock trials with the TE of shock trials, where the TE between fish in WT-WT pairs was measured. When we considered the TE of merged ethogram,^17^ which consists of the signs of change in the forward speed and turn, we found a significant difference (Figure 3M) between no-shock and shock conditions (*p* = 0.04003, effect size = 0.87837). Next, rather than the sign of change, we calculated TE from the forward speed only (see STAR Methods) and found that the TE of speed increased significantly (Figure 3N) in shock trials (*p* = 0.02116, effect size = 0.92814). Moreover, the ΔTE, which is the net flow of TE between fish, was also significantly higher in the shock trials (Figure 3O, *p* = 0.01519, effect size = 1.30230). However, no significant difference could be observed when we calculated TE from only the turning data (Figure 3P, *p* = 0.15481).

### Transgenic fish (TG) show a decrease in the attack posture rate while WT-TG pairs show a decrease in the near rate and an increase in mutual distance

**Figure 4:**
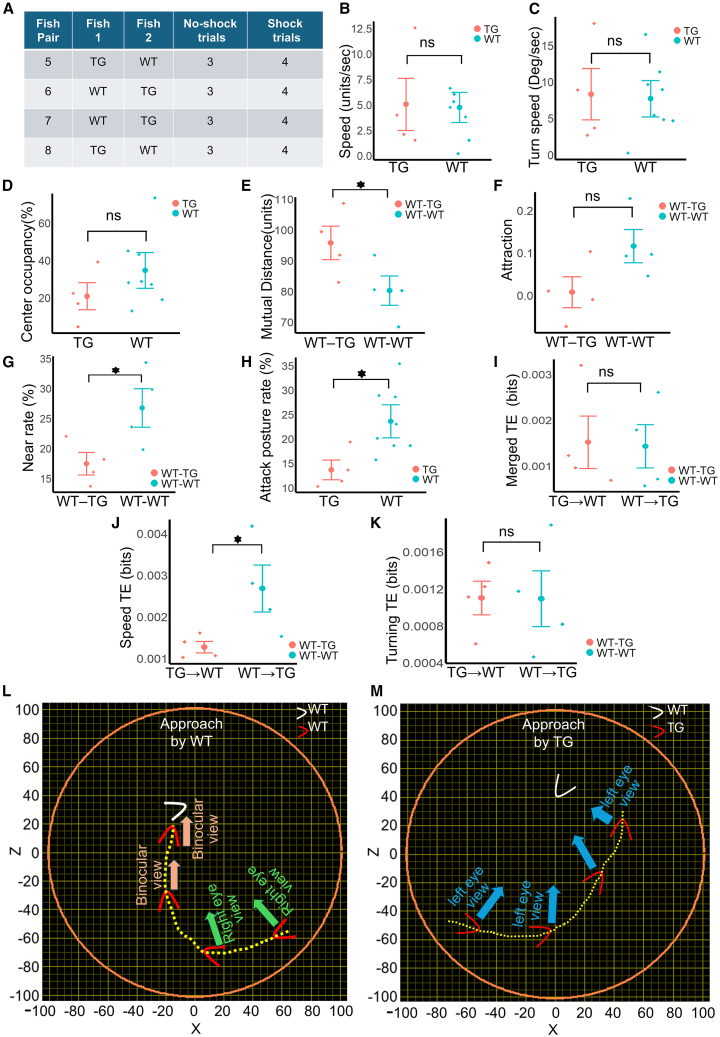
Various comparisons between WT-TG and WT-WT pairs, as well as between WT and TG fish Throughout this figure, red and blue denote either TG and WT fish, or WT-TG pairs and WT-WT pairs, respectively. Enlarged points at the center of the vertical red and the blue lines denote mean values, and the two ends represent error bars (mean ± 0.5∗standard deviation). Individual fish or pairs are denoted as single data points. (A) The number of no-shock and shock trials in four pairs of zebrafish where TG fish were paired with WT fish. Shock trials were conducted after all no-shock trials finished. Trials corresponding to one pair were all performed on the same day. (B) Comparison of forward speed between WT and TG fish. (C) Comparison of the turning speed between WT and TG fish. (D) Center occupancy rate of WT and TG fish. (E) Mutual distance compared between WT-WT and WT-TG pairs. (F) Comparison of mutual attraction between WT-WT and WT-TG pairs. (G) Comparison of the near rate between WT-WT and WT-TG pairs. (H) Attack posture rate of WT and TG fish. (I) Comparison of TE flow between WT and TG fish, where TE (WT to TG and TG to WT) was calculated from the merged ethogram. (J) Comparison of TE when only forward speed is considered. (K) Comparison of TE when only turning speed is considered. (L) Example of one successful approach made by a WT fish to its paired opponent. Red and white arrowheads represent the position and orientation of the approaching fish and the target fish, while the blue, green, and pink arrows represent left eye view, right eye view, and binocular view of the target, respectively. (M) Example approach made by a TG fish to its WT opponent, which eventually ends in failure to bite.

Four transgenic (TG) fish with the impairment of the dorsal Hb-IPN winner’s circuit (silenced dHbL-d/iIPN pathway) were paired with four WT fish to conduct three no-shock and four shock trials for each pair (Figure 4A). To make a comparison between the WT and TG fish, we used the average values from eight WT fish (eight WT fish from the previously mentioned four WT-WT pairs) and four TG fish (from four WT-TG pairs), as each of these fish was paired with one WT fish. The four WT fish paired with the four TG fish were not considered in the WT-TG comparison, as their opponents were TG fish, which could have influenced their behavior, possibly in a different manner. Statistical comparisons between the WT and the TG fish, or the WT-WT pairs and the WT-TG pairs, were conducted using the Wilcoxon Rank-Sum test. A video containing the continuous plots of HDs and trajectories of a WT-TG fish pair is shown in Video S3, where the TG fish is marked in red. We investigated differences between the WT and the TG fish (eight WT vs. four TG) as well as between the WT-WT pairs and the WT-TG pairs (four WT-WT pairs vs. four WT-TG pairs). First, we looked at the forward speed and turning speed of WT and TG fish in VR. The forward speed was not significantly different between the WT and the TG fish in both no-shock and shock conditions (*p* = 0.35542 for no-shock trials (figure not shown) and *p* = 0.46616 for shock trials, Figure 4B). Similarly, no difference was observed in turning speed.

Video S3. Example trajectories of a WT and a TG fish from one WT-TG pair The TG fish is marked in red, while the opponent WT fish is shown in white. The speed of the video is 3× of the original.

(*p* = 0.48251 in no-shock trials (figure not shown) and *p* = 0.39945 in shock trials, Figure 4C). Center occupancy rate did not differ significantly between the WT and the TG fish in both shock condition (*p* = 0.10137, effect size = 0.75568, Figure 4D) and no-shock condition (*p* = 0.1883, figure not shown). A reduction in mutual distance was observed in the WT-WT pairs compared with the WT-TG pairs in shock trials (*p* = 0.0329, effect size = 1.23049, Figure 4E) but not in no-shock trials (*p* = 0.5122, figure not shown). However, attraction was not significantly higher in WT-WT pairs in the shock condition (*p* = =0.09697, Figure 4F) as well as the no-shock condition (*p* = 0.27937, figure not shown). Compared to WT-WT pairs, the near rate decreased significantly in WT-TG pairs (Figure 4G, *p* = 0.02830, effect size = 1.33273) in shock trials but not in no-shock trials (*p* = 0.43885, figure not shown). Moreover, the attack posture rate was significantly higher in WT fish in shock (*p* = 0.01672, effect size = 1.31164, Figure 4H) condition, though similar differences were not found in the no-shock condition (*p* = 0.20267, effect size = 0.69140).

### Movement of the TG fish was influenced more by the WT fish

To investigate how the WT and the TG fish influence each other’s movement, we calculated values of information flow from WT to TG (*TE*_*WT→TG*_) and from TG to WT(*TE*_*TG→WT*_) fish in both no-shock and shock trials. TE of merged ethogram,^17^ which is calculated from the signs of the change of speed and turn over successive frames, did not significantly differ in either the no-shock (*p* = 0.43073, figure not shown) or the shock (*p* = 0.44262, Figure 4I) trials. However, a significant increase in *TE*_*WT→TG*_ was observed when TE is calculated from only the forward speed in shock trials (Figure 4J, *p* = 0.03490, effect size = 1.31697), though no significant difference was observed in no-shock trials (*p* = 0.36296, figure not shown). On the other hand, no significant difference could be observed in TE calculated from only turning speed, in either the no-shock (*p* = 0.52613, figure not shown) or the shock trials (*p* = 0.44262, Figure 4K).

While analyzing biting behavior, we found that the average number of bites scored by the WT and the TG were similar (8.24 bites by WT fish and 7.53 bites by TG fish per trial, no statistical difference). Trial-wise number of bites is shown in Table S2. Next, considering the ratio of bites and approaches (bite per approach) as the probability of scoring a bite from an approach, we compared the bite per approach values of the WT and the TG fish. As shown in the Figure S3, the bite per approach ratios of the WT fish were higher than the TG fish (Wilcoxon Rank-Sum test, *p* = 0.03727, effect size = 1.06535). This difference in biting skill in VR prompted us to investigate how both WT and TG fish viewed their paired opponent. Besides the previously used FBVP, LEP, and REP, we also used the left eye index (LEI, see STAR Methods) to check if there is any difference in the usage of the left and the right eye in WT and TG fish. First, to visually inspect the lateral bias in eye-usage during approaches, we observed the approaches made by the WT and the TG fish. Examples of approaches are shown in Video S4. One example of approaches made by the WT fish and the TG fish is shown as trajectories plotted in Figures 4L and 4M, respectively. In case of the approach made by the WT fish (Figure 4L, denoted by red arrow and dotted line to show the heading direction and trajectory, white arrow is the opponent WT fish), initial view of the opponent was with the right eye, which was succeeded by a change of course to bring the opponent within the binocular view field until the bite-collision occurred. On the other hand, in the approach made by the TG fish (Figure 4M, TG as red arrow, WT as white arrow), the initial view of the opponent was with the left eye, and throughout the approach, the TG fish failed switch to the binocular view toward the opponent fish, and could not reach the opponent to make a bite.

Video S4. Movies of examples of approach trajectories Left video: Examples of approach episodes made by WT fish. Values of LEI, LEP, BVP, and REP are displayed in the uppermost section of the video. Trajectories of two fish are shown within the circle corresponding to the VR arena, with each fish shown as a triangle, with the tip of the triangle corresponding to the head. The red triangle denotes the approaching fish. The use of the left eye, the right eye, and the binocular vision of the approaching fish is shown in the LEDs on the fish body depicted below. Right video: Examples of approaches made by TG fish. The video speed is the same as the original.

### Between-group analysis shows significantly less percentage of the field of binocular view as well as significantly higher left eye usage in TG fish

**Figure 5:**
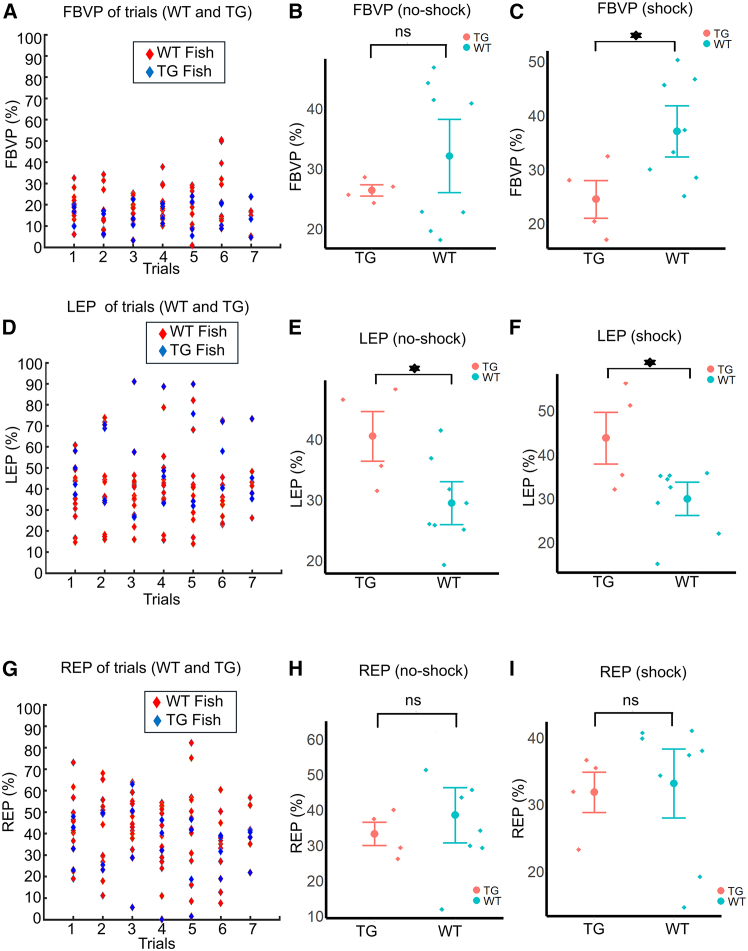
Comparison of the FBVP, LEP, and REP between WT and TG fish (A) FBVP of all trials by WT and TG fish. Red dice: WT fish. Blue dice: TG fish. (B) Comparison (WT vs. TG) of FBVP in the no-shock condition. (C) Comparison (WT vs. TG) of FBVP in shock condition. (D) Comparison of the LEP of trials by WT and TG. (E and F) Comparison of LEP (WT vs. TG) in no-shock (E) and shock (F) conditions. (G–I) Similar comparison was performed for the REP of WT and TG fish.

To investigate if there is a higher reliance on any specific eye in WT and TG fish, we compared the FBVP, LEP, and REP (see STAR Methods) between the WT and the TG group in approaching episodes extracted from no-shock and shock trials separately. First, the FBVP of individual trials is shown in Figure 5A. WT fish did not show higher FBVP than TG fish while considering data from the no-shock condition (Figure 5B, *p* = 0.53384, effect size = 0.56239). On the contrary, FBVP was significantly higher in WT fish during the shock condition (Figure 5C, *p* = 0.0254, effect size = 1.20799). Next, the LEP of WT and TG fish calculated over the whole trials is plotted in Figure 5D.TG fish had higher LEP than WT fish in approaches extracted from both no-shock (Figure 5E, *p* = 0.03727, effect size = 1.23337) and shock (Figure 5F, *p* = 0.0314064, effect size = 1.25981) trials. Regarding REP, trial-wise REP values of WT and TG fish are displayed in Figure 5G. Contrary to the LEP and the FBVP, no significant difference in REP could be detected between the WT and the TG fish in approaches extracted from either the no-shock (Figure 5H, *p* = 0.22235) or the shock trials (Figure 5I, *p* = 0.17512).

### FBVP increases while distance to the opponent decreases, and WT fish have a higher FBVP in proximity to the opponent

**Figure 6:**
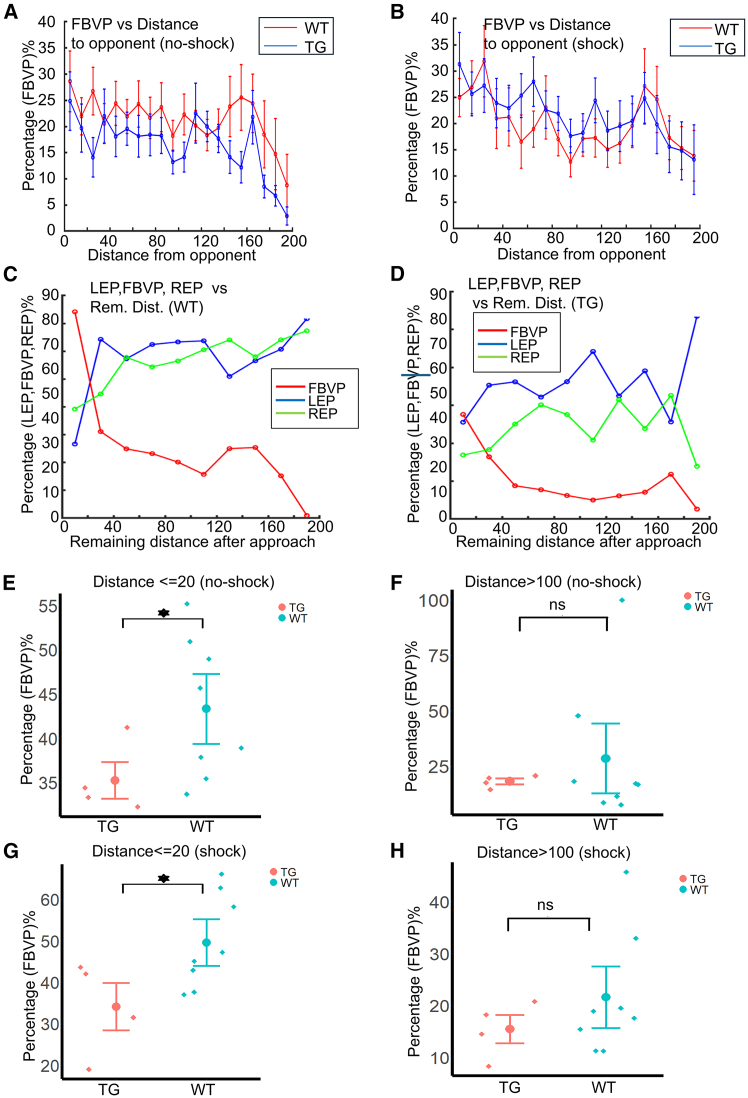
Change of LEP, FBVP, and REP with respect to the distance from the opponent fish (A) Plots of mean FBVP with respect to distance bins (20 bins, distance 0–200) for WT and TG fish (red line: WT fish, blue line: TG fish) in the no-shock trials. Error bars (mean ± 0.5∗standard deviation) are shown over the fish-averaged mean FBVP at each distance bin. (B) Same as (A), with data from shock trials. (C) Plots of the mean LEP, FBVP, and REP with respect to the remaining distance in approaches. Data are pooled from all WT fish. (D) Same as (C), with data pooled from all TG fish. (E) Comparison of FBVP between WT and TG fish in case the remaining distance at the end of approaches was ≤20 units (high proximity). (F) Same comparison as (E), when the remaining distance of approaches was >100 units (low proximity). (G and H) Similar comparison of FBVP between WT and TG fish is shown in (E) and (F), though for data in the shock condition.

As we have found that FBVP increases significantly in WT fish compared to TG fish, we wanted to check if FBVP also depends on distance from the opponent fish. To have a quick glance at this, we first pooled all the frames where the opponent fish was within the binocular view field, as well as the distance with respect to the opponent in those frames. Next, the mean FBVP was calculated for distance bins (20 bins of distance 10, distance range 0–200) with data from all WT fish and all TG fish groups separately, and further divided into data from no-shock and shock trials within the groups. The groupwise means of FBVP with error bars (showing the spread of ±0.5 standard deviation) are shown for data from the no-shock (Figure 6A) and the shock trials (Figure 6B). In the no-shock condition, FBVP of WT fish was not significantly different (*p* = 0.33556) than TG fish when the distance to the opponent fish was less than 100 units, or when the distance was more than 100 units (*p* = 0.27611). On the other hand, in the shock condition, FBVP was statistically higher in WT fish when mutual distance was less than 100 units (*p* = 0.01093, effect size = 1.42749) but not when greater than 100 units (*p* = 0.22235). Next, rather than considering all frames, we gathered frames corresponding to approaches only and looked for frames where the opponent fish was within the binocular view field, separately for the WT and the TG groups. Then we calculated the mean FBVP for the same 20 distance bins while also recording the remaining distance to the opponent at the end of approaches. Besides calculating the mean FBVP, we also calculated the mean LEP and the mean REP in the same way. The mean LEP, FBVP, and REP of the WT and the TG group are shown in Figures 6C and 6D, respectively, where the FBVP (red line, Figures 6C and 6D) seems to increase with increased proximity to the opponent. To compare FBVP of the WT and the TG group further with respect to the proximity to the opponent, we then compared the FBVP of the WT and the TG fish in two cases: 1) high proximity to opponent: when the remaining distance at the end of the approaches was less than or equal to 20, which is a value just smaller than the body length of the 3D model of zebrafish in VR (25 units), and 2) low proximity to the opponent: when the remaining distance at the end of the approaches was greater than 100, which is the radius of the VR arena. Furthermore, we did the above comparison in the case of approach data taken from the no-shock and the shock trials separately. We found that in the case of the no-shock data, FBVP was higher in WT fish in the high proximity case (Figure 6E, *p* = 0.03727, effect size = 1.04082) but not in the low proximity case (Figure 6F, *p* = 0.27611). Similarly, in the case of shock condition, FBVP was higher in the WT fish when proximity was high (Figure 6G, *p* = 0.03727, effect size = 1.16896) but not when proximity was low (Figure 6H, *p* = 0.28432) to the opponent. These results imply that while approaching proximity to the opponent, the WT fish could place the opponent within the field of binocular view with greater ability.

### Left eye index (LEI) was significantly higher in the TG fish

**Figure 7:**
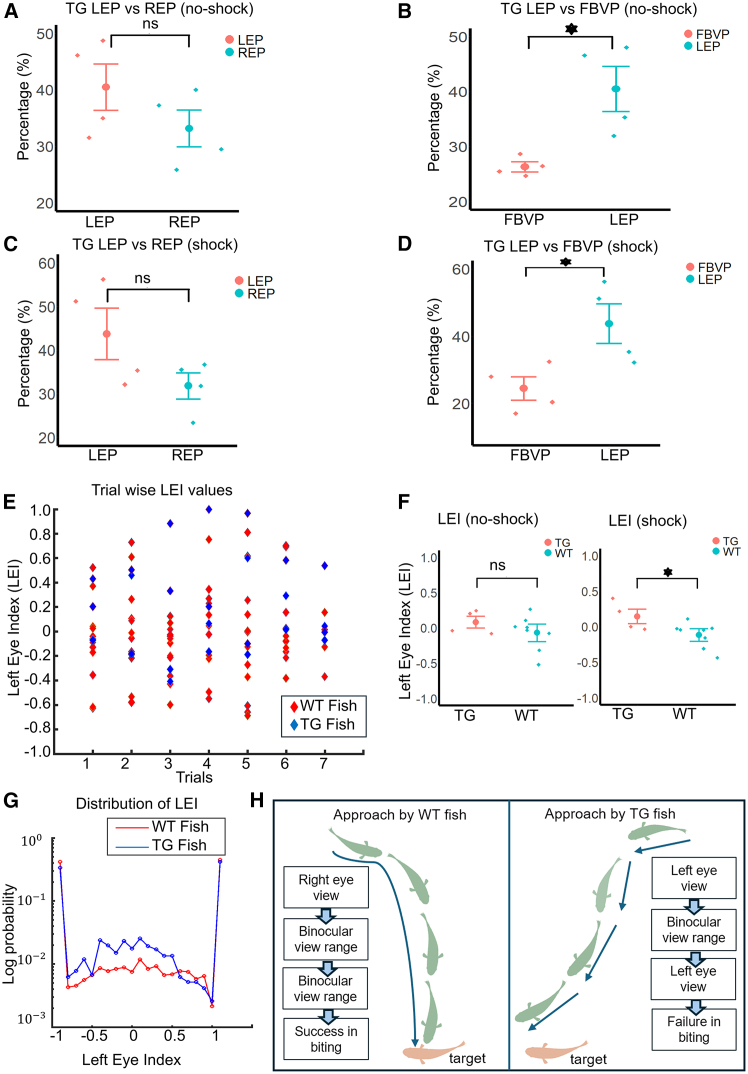
Within-TG-group comparison of LEP, FBVP, and REP, as well as results of the analysis of the left eye index (LEI) (A) Comparison between LEP and REP within the TG fish during the no-shock condition. (B) Comparison between LEP and FBVP within the TG fish during the no-shock condition. (C) Comparison between LEP and REP within the TG fish during the shock condition. (D) Comparison between LEP and FBVP within the TG fish during the shock condition. (E) Values of LEI of all trials from WT fish and TG fish. Red dice: WT fish. Blue dice: TG fish. (F) Left: Comparison of LEI values between WT and TG fish in the no-shock condition. Right: Comparison of LEI values between WT and TG fish during the shock condition. (G) Distribution of the LEI values of WT fish (Red line) and TG fish (Blue line). The vertical axis corresponds to log probability. (H) Left: Conceptual schematic of an approach by WT fish (green) to a target fish (pink), with the blue arrow(s) showing head direction. Target is observed initially with the right eye, though a subsequent switch to binocular vision is maintained until collision. Right: Schematic of an approach by TG fish (green). Approach starts with the left eye view of the target, but the TG fish was unable to maintain binocular vision and switched again to left eye view, causing it to miss the target.

To investigate the use of individual eyes as well as the binocular view field, we analyzed and compared the LEP, FBVP, and REP of the WT and the TG fish. The within-WT group analysis done on the approaching episodes showed that there is no significant difference in the LEP vs. REP and the LEP vs. FBVP comparisons in either the no-shock trials or the shock trials. On the other hand, in TG fish, similar analysis revealed that though the LEP was not significantly higher than REP (Figure 7A, *p* = 0.29194), it was higher than FBVP (Figure 7B, *p* = 0.0153356, effect size = 1.51088) in the no-shock condition. Furthermore, similarly in the shock condition, no significant difference was found between LEP and REP (Figure 7C, *p* = 0.29194), but LEP was significantly higher than FBVP (Figure 7D, *p* = 0.0307853, effect size = 1.41009). These results demonstrate the predominant use of the left eye in TG fish in both no-shock and shock conditions.

Next, we used the LEI (see STAR Methods) to check whether there is a difference between the WT and the TG fish in terms of reliance on either the left or the right eye. Figure 7E shows the LEI values of individual trials (both no-shock and shock trials) of WT and TG fish, with each LEI value representing one trial. Showing a higher reliance on the left eye, LEI was significantly higher in TG fish (Figure 7F(right), *p* = 0.02540, effect size = 1.20166) in the shock condition but not in the no-shock condition (Figure 7F(left), *p* = 0.33556).

While we observed the approaches made by the WT and the TG fish, we found that compared to the WT fish (left video, Video S4), TG fish had more approaching episodes (right video, Video S4) where the LEI values (shown at the top of the video) were within the range of −0.5∼0.5. This implied the increase of alternating use of the left and the right eye within the same approach in TG fish. The log probability distributions of LEI values are shown in Figure 7G, where both WT and TG fish show the tendency to view the opponent fish during approach with solely the left or the right eye, resulting in high probability of LEI values of −1 and 1. Please note that the binocular view field, in which the opponent could be viewed with both eyes simultaneously, is not considered in the calculation of LEI. While focusing on the middle part of the graph, that is, for LEI values within the range −0.5∼0.5, the sum of probability of LEI values in the TG fish (blue line, Figure 7G) was 2.3 times that of WT (red line, Figure 7G). The LEI value range of −0.5∼0.5 is from approaches in which the opponent fish was observed with the right and the left eye in an alternating manner. We think viewing the opponent fish with both the left and the right eye alternatingly within the same approach made it more difficult to reach the opponent to score a bite.

A conceptual Figure 7H shows the summary of the difference between the WT and the TG fish. The schematic of the left panel shows an example of an approach made by a WT fish to its target. In this example, at the beginning of the approach, WT fish identifies the target with its right eye and then modifies the body angle to place the target within the binocular view field until it collides with the target to cause a bite event. On the other hand, as shown in the right panel, the TG fish starts the approach by viewing the target with its left eye, and though a switch to the field of binocular view occurs briefly, it is not sustained in proximity of the target, causing the TG fish to be unable to hit the target. We think this phenomenon is due to the silenced dHbL-d/iIPN pathway in the TG fish, which is discussed in the next section.

## Discussion

With the aim of investigating social interaction, we have designed a VR system that facilitates interaction between two adult zebrafish with the teleportation of live movement to realistic-looking 3D avatars in real time. To investigate the neural mechanisms of dyadic fish fight by *in vivo* optical imaging, we attempted to reproduce the fighting behavior in our VR system by introducing a novel electrical stimulus paradigm. In this paradigm, avoidance of the periodic stimuli required the fish to bite the opponent, while high transient shock was used to provide feedback for biting. Due to the head-fixed condition and the slower speed of movement in VR, we did not observe the typical real-world fighting behavior such as circling around each other at high speed. However, we still observed the bite-like behavior in which zebrafish moved toward the opponent fish to hit the mid part of the body. We expect that our proposed VR system can be a platform for subsequent imaging studies that can reveal the specific mechanism of neural processing during various forms of social interaction.

Our VR system also enables the investigation of how zebrafish view each other in a novel environment. Instead of measuring the actual eye movement of the fish, we used the information of the HD inside VR and calculated where the opponent fish is within certain angular ranges, defining the left, right eye view, as well as the binocular view field. In many typical episodes of approaches that ended in bite-like behavior, the avatar of the target fish was initially observed with the left or right eye view, which was subsequently followed by a change in HD in the VR to bring the target fish within the binocular view field that lasted until the end of the approach. In WT pairs, we found no bias in either the left or the right eye view, and the opponent was placed within the binocular view field as much as either of the individual eyes.

We discovered that the presence of electrical stimulus caused reduced mutual distance and increased attraction, as well as increased flow of information between fish in the shocked trials. Furthermore, we found that the FBVP increased in the shock trials. These findings suggest that the introduction of mild electric stimuli may have provided further motivation to the fish to bite the opponent and avoid the periodic shock. Nevertheless, spontaneous biting behavior was observed in no-shock trials as well, which we think hints at the innate inclination of adult male zebrafish toward dyadic fight or some kind of affiliative interaction with a conspecific male. In our view, this innate behavioral tendency was further enhanced in the shock trials due to electric stimulation.

In our previous work,^30^ we found that the TG fish with the silenced dHbL-d/iIPN pathway struggled to perform a directional task that required the egocentric distinction of right and left. Other literature^33^ indicates that activity in the Hb contributes to optimized prey capture performance in fish that are experienced during preying. The dorsal Hb in teleosts is homologous to the mammalian medial Hb and has been shown to modulate fear responses and social conflict outcomes in adult zebrafish.^23^^,^^25^ Taken together, it can be inferred that the signal from the Hb along the dHbL-d/iIPN pathway plays a role in integrating optical cues from both eyes to form binocular vision, which is necessary for prey capture and dyadic fight, and any disruption of the signal due to the impairment of this pathway may cause imprecision in the zebrafish’s attacking movement. In this work, we used the dHbL-d/iIPN pathway-silenced TG fish to pair with the WT fish to investigate if our VR system can reveal any deficiency in the specific behavior of this transgenic line. We found that the TG fish showed a lower bite-to-approach ratio than the WT fish. We found that the LEP of TG fish was significantly higher than that of WT fish. On the other hand, TG fish used significantly less binocular vision, and the frequency of using the left and the right eye alternatingly within the same approach was higher in the TG fish. We can assume that due to the impairment of the dorsal Hb to IPN pathway, the TG fish found it harder to form binocular vision, which is probably important for prey capture^35^^,^^36^ in rotating the body axis toward the prey. Due to the higher degree of presence of LEI values within the range of ±0.5, as well as the reduced binocular vision, TG fish found it more difficult to reach the paired opponent. Our results showing the inverse relationships between the FBVP and the remaining distance support the above finding.

As we have performed all our experiments in a head-fixed VR condition, we also opted to investigate whether we could observe our findings regarding the reduction in the use of the binocular view field in TG fish in a real-life scenario. As explained in the Table S3, with the analysis of videos of real-life dyadic fights (Videos S5, S6, and S7) between WT and TG fish,^37^ we found that even in real life, the TG fish showed reduced use of the binocular view field, which validates our finding in VR.

Video S5. A 10-min long video of a real-world dyadic fight between one WT (ID: 6BC) and one TG (ID: 20D) fish

Video S6. A 10-min long video of a real-world dyadic fight between one WT (ID: 17D) and one TG (ID: 8 TC) fish

Video S7. A 10-min long video of a real-world dyadic fight between one WT (ID: 13D) and one TG (ID: 2 TC) fish

The Hb and IPN in zebrafish have been assigned to various functional roles. For example, in case of encountering a robot with aggressive predator-like approaches, week-old larval zebrafish could learn avoidance, during which several brain areas, including Hb, were activated, and an ablation of Hb caused significant impairment in threat learning.^38^ It was also shown that while juvenile zebrafish can form long-term memories after multiple days of conditioned place avoidance (CPA) training, the ablation of dorsolateral habenula (dlHb) in this animal causes failure to adapt to a new learning rule.^39^ Furthermore, forebrain regions such as the Hb were shown to have greater activity during prey capture in experienced zebrafish (33, and the ablation of the habenula causes a reduction in prey capture. These findings elucidate the involvement of the Hb in innate functions that are required for the survival of the animal. On the other hand, the IPN has been found to be a site for the integration of the heading signal from the anterior hindbrain with visual information,^40^ and heading direction is represented in the IPN in a topographical column structure. Other recent works found that HD cells exist in the anterior hindbrain of zebrafish,^41^ and the lateralized projection from the Hb to the IPN is required for landmark tracking in visual scenes,^42^ possibly through a Hebbian-type strengthening of the synapses between Hb neurons that fire when landmarks appear within their visual receptive fields, and HD cells in the d/IPN that respond to specific directions. In our study, we think it is possible that the avatar of the opponent fish also acts as a landmark for the approaching fish, and the connectivity between Hb to the HD cells in IPN may be hampered in the TG fish due to the silencing of the dHbL-d/iIPN pathway, thus making it more difficult for the TG fish to make effective approaches toward the opponent. We assume that one of the functions of this pathway is to consolidate the visual target information in accordance with the activities of Hb cells and HD cells of the IPN and initiate the mechanism of rotating the body axis to point at the target so that the target can be brought within the binocular view field prior to the final part of the attack.

Lateralization in behavior, that is the preferred use of one eye in specific tasks, has been reported across species including zebrafish,^43^^,^^44^^,^^45^ in which the right hemisphere (left eye) is dominant in the discrimination of social companions and individual or familiarity-based recognition, while the left hemisphere (right eye) is specialized for the discrimination among categories such as conspecifics and heterospecifics. About the behavioral lateralization in zebrafish, it has been suggested^46^ that the right hemisphere in zebrafish, which controls the left eye, is used more when escape from danger is being planned. Left eye view in zebrafish is also believed to be involved in assessing the differences between familiar and unfamiliar objects, as well as the visual inspection of the environment, and to fixate on social companions,^47^^,^^48^ whereas decisions to attack prey or targets in a social context are made more with the right eye.^46^ The left-right information is wired asymmetrically in a zebrafish’s brain, where the left-side Hb projects to the dorsal part of the midbrain’s interpeduncular nucleus (IPN), and the right-side Hb projects to the ventral IPN. This laterotopic representation is established through the transmembrane protein Cachd1, which is required for neurons^49^ in the Hb to acquire left-right asymmetric character. Moreover, left-right asymmetry in the Hb is required^50^ for it to respond to both visual and olfactory stimuli by processing each type of information differently and sending it to distinct brain regions. In zebrafish, the left Hb is more responsive to visual stimuli, sending information to the dorsal interpeduncular nucleus (dIPN), while the right Hb is more responsive to olfactory stimuli, sending information to the ventral interpeduncular nucleus (vIPN). It has also been found that left dHb, but not the right, is the primary mediator of light pref.^51^ as it contains light-responsive neurons that are responsible for mediating light-preference behavior in zebrafish larvae, and a mutant fish with aberrant Hb function in the left dHb^52^ resulted in a lack of light preference. We assume that due to the asymmetry of the dHbL and dHbM between the left and right dHb (Figures 1A and 1B) as well as the asymmetry in the visual information processing, the ablation of the dHbL-d/iIPN pathway caused greater impairment of the left dHbL-d/iIPN pathway than the right one in the TG, which resulted in a greater dependence on the right Hb and therefore the left eye view in the TG fish. Furthermore, the increased alternative use of the left and the right eye during approaches made by TG fish is also consistent with our previous finding that this TG fish possessed impaired directional (left-right) information processing during decision making. As part of the visual target processing and physical initiation to start an approach, the dHbL-d/iIPN pathway probably plays an important role in coordinating the information from two eye systems to form binocular vision. Our VR system can thus provide valuable insights into investigating behavioral abnormalities in mutants with certain blocked brain regions or pathways, as well as in model animals for neurological disorders.

### Limitations of the study

This study has generated a VR-based platform to facilitate interaction between multiple adult zebrafish in a head-fixed state. While this methodology is designed with the aim of investigating the corresponding brain activities with simultaneous use of a multi-photon microscope, such optical imaging studies are beyond the scope of this paper. About hardware used in the study, we think that more state-of-the-art display systems, such as bending organic displays and providing somatosensory feedback using water flow or vibrators, could be added to make a more realistic VR system.

### Conclusion

In order to set up a platform for the investigation of social interaction in zebrafish, we have proposed a VR system that enables simultaneous optical imaging of adult zebrafish. As proof of concept for this system, we have tried to instigate dyadic fights between zebrafish in VR with a newly devised electric shock protocol. We conclude that some features of real-world fish fight (such as biting) can be preserved even in VR, and our system can be effective in invoking such social interactions as well as finding certain behavioral abnormalities in transgenic fish, such as the dHbL-d/iIPN pathway-silenced fish we investigated. Further imaging studies based on our system can reveal new insights regarding how the zebrafish brain encodes social interaction.

## Resource availability

### Lead contact

Further information and requests for resources and reagents should be directed to and will be fulfilled by the lead contact, Hitoshi Okamoto (hitoshi.okamoto@riken.jp).

### Materials availability

This study did not generate any reagents.

### Data and code availability

• All behavior data reported in this study are available in the following public repository: https://doi.org/10.5281/zenodo.16809628.
• All programming codes used to create virtual reality for social interaction are available with the following https://doi.org/10.5281/zenodo.16809628.
• Any additional information required to reanalyze the data reported in this paper are available from the lead contact upon request.

## Acknowledgments

This work was supported by MEXT KAKENHI Grant-in-Aid (H.O., 21H0814, 22H05520, and 23H04976) and by the internal grant of the RIKEN-Kao Collaboration Center.

## Author contributions

Conceptualization, T.I. and H.O.; methodology, T.I. and M.T.; software, T.I.; formal analysis, T.I.; investigation, T.I.; resources, M.T. and Y.T.; writing – original draft, T.I.; writing – review and editing, T.I. and H.O.; funding acquisition, H.O.

## Declaration of interests

The authors declare no competing interests.

## STAR★Methods

### Key resources table

**Table:** 

REAGENT or RESOURCE	SOURCE	IDENTIFIER
**Software and algorithms**		

LabVIEW	National Instruments	https://www.ni.com/en.html
MATLAB	MathWorks	https://www.mathworks.com/
Unity Engine	Unity	https://unity.com/
Visual Studio	Microsoft	https://visualstudio.microsoft.com/
Maya 2008	Autodesk	https://www.autodesk.com/products/maya/overview

### Experimental model and study participant details

All experimental procedures, including the generation of transgenic zebrafish, were reviewed and approved by the RIKEN Safety Management Division Bio Safety and Ethics Section (Protocol number: W2023 – 125, Approval number: 2024-014(1)). The wild-type and transgenic zebrafish were bred and maintained at the Animal Care facilities of the RIKEN Center for Brain Science. This study used adult wild-type zebrafish (RIKEN-Wako, Saitama, Japan) and transgenic zebrafish line *Tg(narp:GAL4VP16); Tg(UAS: tetanus neurotoxin light chain (TeTxLC))*. Fish were maintained in 7-L tanks with continuous water exchange at 28.5°C under the 14-h light/10-h dark cycling, and male adult fish aged more than 3 months were used for the experiments. The description of experiments, animals, results, methods and statistical analysis meet the ARRIVE recommended guidelines 2.0, a checklist of which is also submitted with this manuscript.

### Method details

#### Virtual reality for social interaction

Virtual reality setup for single adult zebrafish was built exactly with the same method previously used.^20^ Adult male zebrafish with age >6 months were head-fixed in the VR apparatus using a stainless rod of radius 0.2 mm, which was attached to the skull with dental bond. Two electrodes were placed on either side of the fish body to provide electrical shock. Two zebrafish were fixed in two identical VR systems that were a couple meters away from each other, and zebrafish could not see each other as each fish was surrounded by LCD screens. In each VR system, a custom control program written in LabVIEW (National Instruments Inc.) and MATLAB (MathWorks Inc.) communicated with scripts written in Unity (Unity Inc.) game engine with TCP/IP. A camera continuously recorded the tail movement of fish, and speed and the head direction change were calculated from the tail movement.^20^ Before the experiment, each fish did adaptation training in their respective VR space alone. After this adaptation of 1 h, fish were put together in the same VR space so that they could interact with each other. To enable this, another control program written in LabVIEW was used, so that both fish could see the 3D model of the opponent in the VR. In this case, the control program calculated the speed and head direction of both zebrafish from their respective tail movements, and communicated with two separate sets of Unity scripts residing in dedicated computers for VR systems 1 and 2. The 3D model of zebrafish that was used was made with MAYA (Autodesk inc.), and skin pigmentation of real zebrafish was used as the skin of the 3D model. Joints in the 3D model were moved by sinusoidal oscillation during swimming in the VR to make it more realistic. Each pair of fish could interact in the VR in trials of 30 min of length. An inter-trial interval of 10 min, when VR displays were switched off, was administered after every trial.

#### Center occupancy rate

In the circular VR arena with a radius of 100 VR units, an invisible inner circle with a radius of 70 VR units is defined as the center area, which had an area half of that of the circular VR arena. The center occupancy rate was defined as the percentage of frames in which the position of the fish was inside the center area.

#### Near rate

Near rate between two fish was defined by calculating the percentage of time frames when the distance between two fish in the VR was less than or equal to 50 units, as shown in the following equation:

(Equation 1) $$Near\;rate=\frac{N\left(distance\leq 50\right)}{N\left(all\right)}\times 100$$

Here, *N*(*distance* ≤ 50) is the number of frames where mutual distance between two fish in the VR space was less than or equal to 50, and *N*(*all*) is the total number of frames within the time period, for example, one whole trial.

#### Attack posture rate

The attack posture rate was calculated in the following way. For one specific fish, we checked how many frames in each trial the fish was facing the avatar of the other fish. In each frame, an extended line was calculated from the mouth of that fish along the direction of the head direction, and we assumed that if this imaginary straight-line went through a circle centered in the middle point of the avatar of the other fish and with a radius of 25 units, then the fish in question had an attacking posture. The percentage of frames in a trial where one fish had attacking posture was called the attack posture rate.

#### Attraction

Attraction between two fish was calculated with a method similar with a previous paper (Larch and Baier, 2018, ref. 11). First the distance *dist*_*real*_ between two fish was calculated over a time-period (a trial) by averaging the distance of all frames within the period. Next, the position time series of one fish was shuffled randomly 10 times, and with each shuffled position time series, mean distance with the other fish (*dist*_*shuffled*_) was calculated. The 10 values of *dist*_*shuffled*_ represented the expected mutual distance by chance. Attraction was defined as the following equation:

(Equation 2) $$Attraction=\frac{mean\left({dist}_{shuffled}\right)-{dist}_{real}}{mean\left({dist}_{shuffled}\right)}$$

Here, *mean*(*dist*_*shuffled*_) is the average of the 10 values of *dist*_*shuffled*_ mentioned above.

#### Transfer Entropy

Transfer entropy (TE)^34^ from a variable *P* to another variable *Q* is the reduction in the amount of uncertainty in future values of *Q* by knowing the past values of both *P* and *Q*, compared to the case when only past values of *Q* are known. The transfer entropy $T_{P\to Q}$ that denotes the flow of information from *P* to *Q* is calculated by the following equation:

(Equation 3) $$T_{P\to Q}=H\left(Q_{t}\;|Q_{t-1:t-L}\right)-H\left(Q_{t}\;|Q_{t-1:t-L},P_{t-1:t-L}\right)$$

Where *H(x)* is Shannon’s entropy of *X* given by:

$$H\left(X\right)=-\sum_{x\in X}p\left(x\right)logp\left(x\right)$$

Where Σ denotes the sum over the variable’s possible values.

The ΔTE, which is the net flow of information between two variables *P* and *Q*, is given by the following equation:

(Equation 4) $$\Delta \text{TE}=T_{P\to Q}-T_{Q\to P}$$

In this study, TE of whole ethogram was calculated according to the method described in Karakaya et al., 2020. For each time point, changes in values of speed and head direction were calculated from the previous and current frame values. An increase from the previous frame was given a positive (+) sign, and a decrease was given a negative (−) sign. The combination of the change of speed and head direction enables the categorization of the whole ethogram into four possible values: (++), (+-), (-+), and (--). TE of the whole ethogram was calculated based on these four possible values. On the other hand, TE of speed and turn were calculated by dividing the value range in the following 5 levels: less than or equal to lowest 5%, between 5% and 35%, between 35% and 65%, between 65% and 95%, and more than 95% of the data. In all cases, a delay of 1 (corresponding to 100 ms) was used for the calculation.

#### Approach detection

To automatically extract episodes of one fish approaching the other in VR, we used the following method. First, we calculated the inter fish distance each frame of a trial. Then we programmatically detected the peaks and valleys in the distance graph. The time lengths from each peak to the next valley were considered for approaches. Within each of such time lengths, we looked for the period when fish A had a higher speed than fish B, and fish was moving toward fish B (fish B was within the ±90^o^ viewing range of fish A). We assigned the episodes that met the above conditions as approaches made by fish A, and vice versa. The distance between the two fish at the end of an approach is used as the ‘remaining distance’ throughout.

#### Left Eye Index

The left eye index is calculated by using the number of left eye views, *N*(*left eye*) and right eye views, *N*(*right eye*) with the following equation:

(Equation 5) $$LEI=\frac{N\left(left\;eye\right)-N\left(rigth\;eye\right)}{N\left(left\;eye\right)+N\left(right\;eye\right)}$$

#### Eye percentage

We created three indices to express how much individual eyes (left and right eye) as well as binocular vision are used to view a target over a period. The Left Eye Percentage (LEP) is calculated from the following equation:

(Equation 6) $$LEP=\frac{N\left(left\;eye\right)}{N\left(left\;eye\right)+N\left(right\;eye\right)+N\left(binocular\right)}$$

Here, *N*(*left eye*), *N*(*right eye*), *and N*(*binocular*) are the number of frames in which target was viewed with the left eye, the right eye and with both eyes respectively.

The Field of Binocular View Percentage (FBVP), which is the percentage of frames where opponent is within the range of binocular vision (±30^o^), is calculated as:

(Equation 7) $$FBVP=\frac{N\left(binocular\right)}{N\left(left\;eye\right)+N\left(right\;eye\right)+N\left(binocular\;range\right)}$$

In similar ways, the Right Eye Percentage (REP) is calculated from the following equation:

(Equation 8) $$REP=\frac{N\left(right\;eye\right)}{N\left(left\;eye\right)+N\left(right\;eye\right)+N\left(binocular\right)}$$

#### Analysis of real-life zebrafish fighting videos

To validate our finding of the reduced binocular vision in the TG fish in real life we have analyzed videos of dyadic fights between WT and TG fish^37^ and visually looked for frames when either of the fish placed the opponent within their field of binocular view. We analyzed three videos of 10 min length where one TG fish is paired with one WT fish (Videos S5, S6, and S7). The frame numbers when binocular vision was used by either fish to view their opponents during approaches are recorded and provided in Table S3.

The size of the tank in the videos is roughly similar to the size of the virtual arena in terms of perception, as the length of the tank is almost eight times the size of a real zebrafish. We observed that in all three videos, the WT fish had a higher number of approaches with the opponent within its FBV (61, 49,43 approaches by WT compared to 24,23,8 approaches by TG, Table S3). This real-life fighting data supports our finding in VR that TG fish had reduced FBVP than WT fish.

### Quantification and statistical analysis

For statistical comparisons mentioned throughout the article, Wilcoxon Signed Rank test was used for comparison between two conditions (no-shock and shock), while Wilcoxon Rank-Sum test was used for statistical comparison between two different groups (between WT and TG fish, or between WT-WT pairs and WT-TG pairs). For both tests, functions provided in MATLAB (MathWorks Inc.) programming language were used. The effect size of statistical comparison was calculated by considering the standardized mean difference between two variables with the following equation:

(Equation 9) $$E=\frac{\mu_{1}-\mu_{2}}{\sigma }$$

Here *μ*_1_ and *μ*_2_ are means of two populations and *σ* is the standard deviation based on both populations. In this work, a *p*-value<0.05 is considered as significant.
