# Supplementary material for: Virtual social interaction reveals that the dorsal habenula-IPN pathway is essential for targeting the opponent
Source: iScience. 2026 Apr 2;29(5):115566. doi: 10.1016/j.isci.2026.115566 (PMC13099522; doi:10.1016/j.isci.2026.115566)
Supplement: Document S1. Figures S1–S3 and Tables S1–S3 [file mmc1.pdf]

iScience, Volume *29*

Supplemental information

Virtual social interaction reveals that the dorsal habenula-IPN pathway is essential for targeting the opponent

Tanvir Islam, Makio Torigoe, Yuki Tanimoto, Ming-Yi Chou, and Hitoshi Okamoto

Supplementary document

Figure S1, S2, S3

Table S1, S2, S3

Video S1-S7 (Video legends: Provided in the main text after the “STAR Methods” section.)

Figure S1: Comparison of forward speed and turning speed

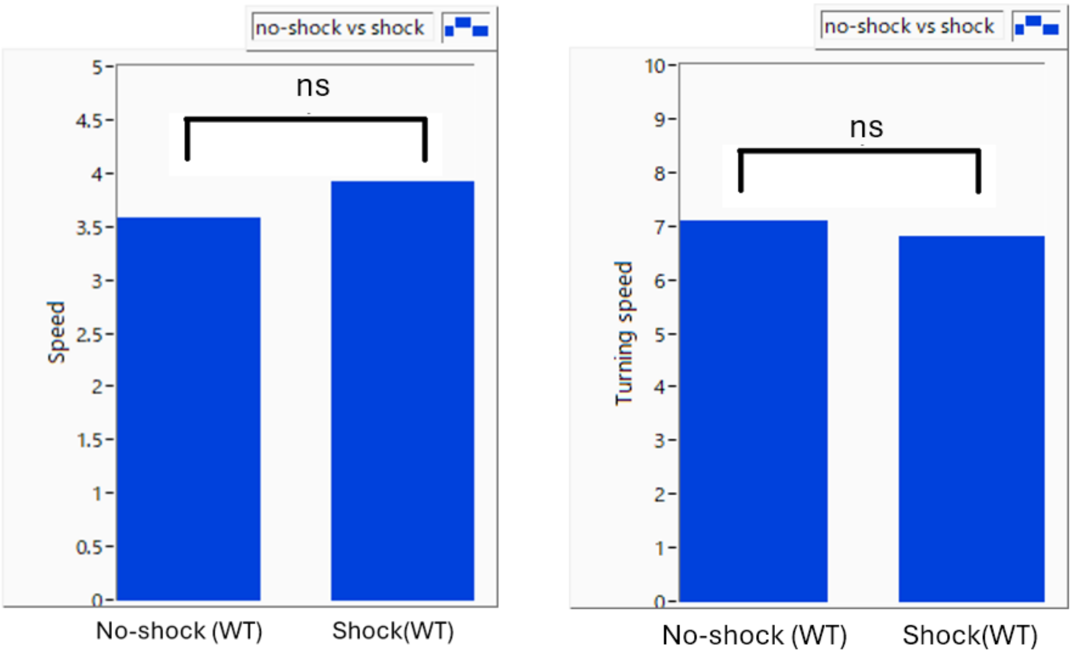

Figure S1: Comparison of swimming speed (left) and turning speed (right) in WT fish during no-shock and shock conditions. No significant difference was observed in either case.

Figure S2: Mutual distance and near rate comparison between actual data and simulated data

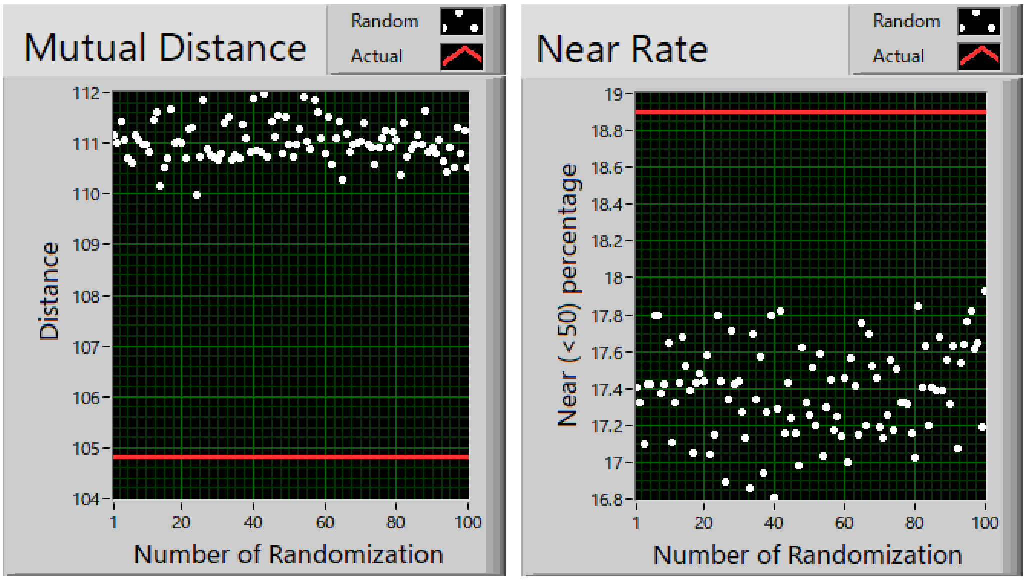

Figure S2-A. Mutual distance (left) and near rate (right) comparison between the actual values (red line) and 100 simulated values (white points). Simulated values are obtained by permutations of the trajectory of one fish at random locations. Trajectory data of the trial#2 of pair#1 is used.

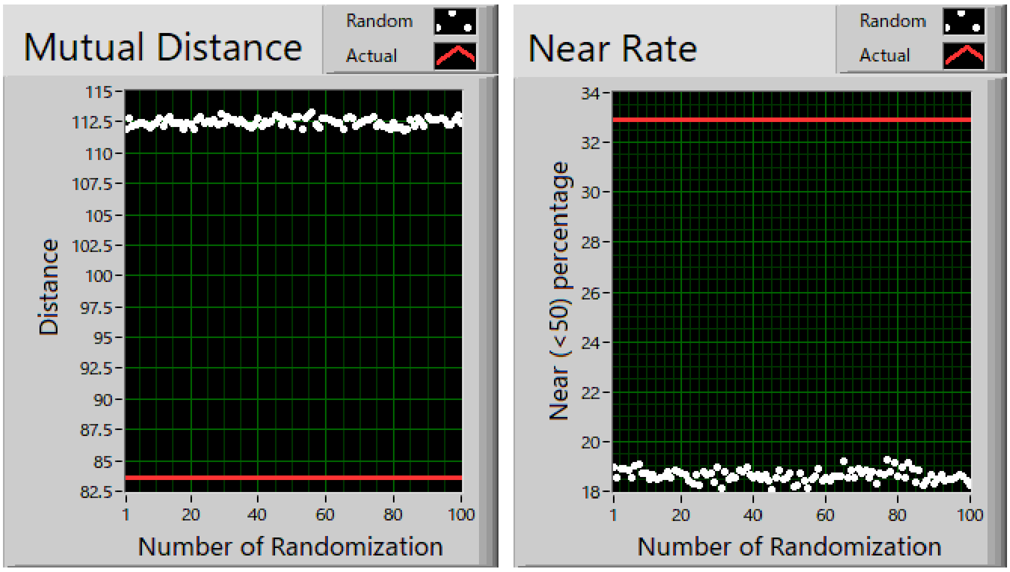

Figure S2-B. Mutual distance (left) and near rate (right) comparison between the actual

values (red line) and 100 simulated values (white points). Simulated values are obtained by permutations of the trajectory of one fish at random locations. Trajectory data of the trial#26 of pair#8 is used.

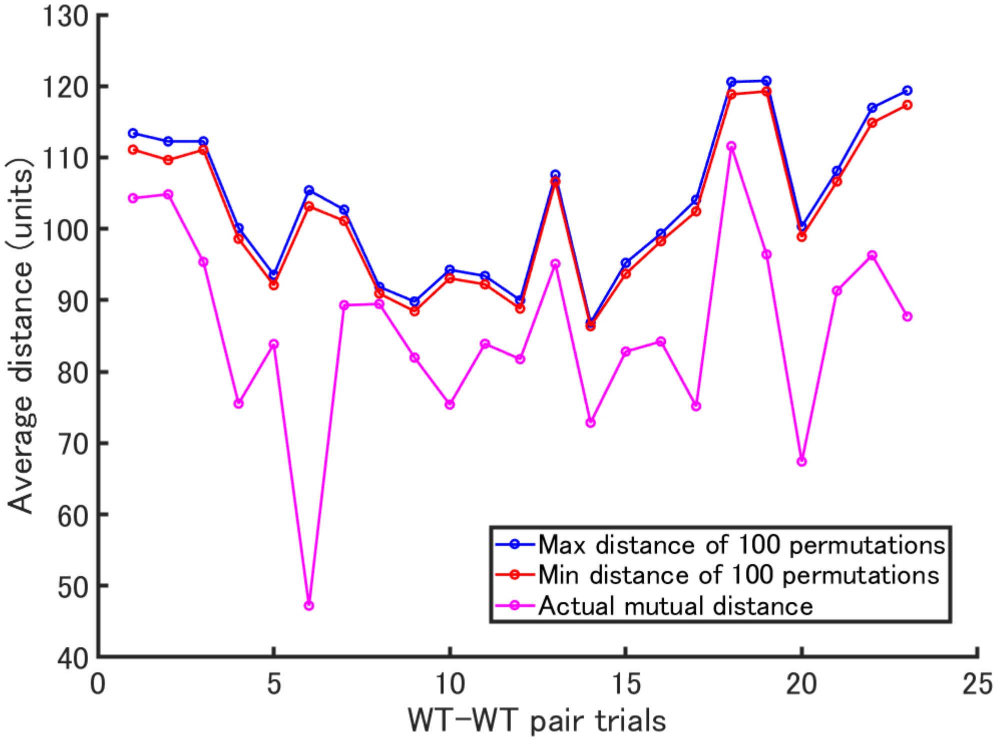

Figure S2-C. Graph showing the average actual mutual distance (Magenta line) of all 23 WT-WT pair trials along with the maximum (Blue line) and the minimum (red line) values obtained from randomly permuting the position of one of the two fish 100 times. It can be observed that the actual distance is less than the minimum of the mutual distance values obtained from the random permutations of position.

Figure S3: Comparison of bite rate (ratio of number of bites and number of approaches) between WT and TG fish

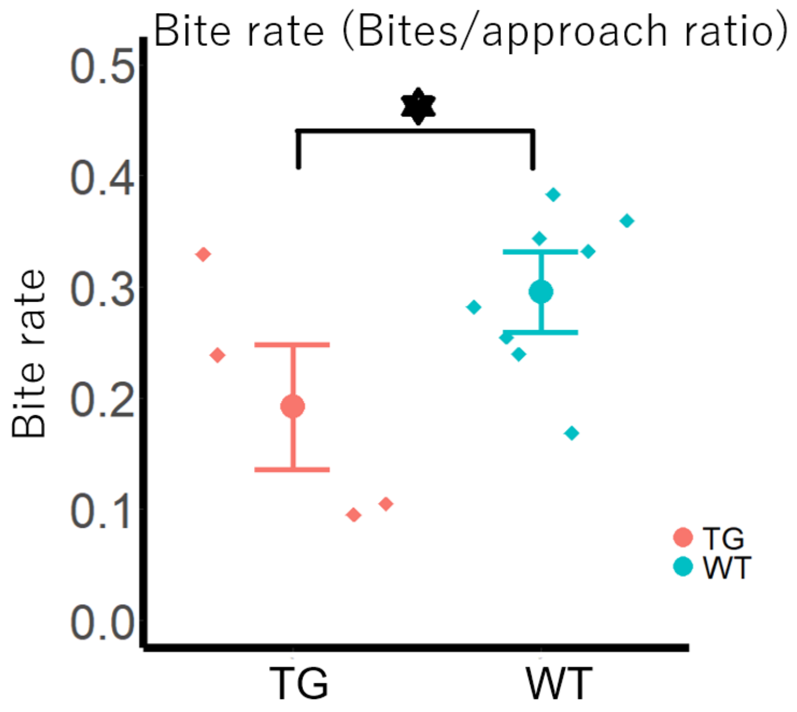

Figure S3: Comparison of the bite rate, which is the ratio of the number of bites and the number of approaches made by each fish, shows that WT fish has significantly higher bite rate than TG fish.

Table S1: Explanation of number of samples used in statistical comparisons in the results section (N-S: No-Shock, S: Shock).

Figure	Comparison	N-samples (data points)	N-samples (data points)
3E	Attack posture rate (WT, N-S vs S)	8 WT fish	8 WT fish
3F	Near rate (WT-WT, N-S vs S)	4 WT-WT pairs	4 WT-WT pairs
3G	Center Occupancy (WT, N-S vs S)	8 WT fish	8 WT fish
3H	Mutual Distance (WT-WT, N-S vs S)	4 WT-WT pairs	4 WT-WT pairs
3I	Attraction (WT-WT, N-S vs S)	4 WT-WT pairs	4 WT-WT pairs
3J	FBVP (WT, N-S vs S)	8 WT fish	8 WT fish
3K	LEP (WT, N-S vs S)	8 WT fish	8 WT fish
3L	REP (WT, N-S vs S)	8 WT fish	8 WT fish
3M	TE-merged (WT, N-S vs S)	8 WT fish	8 WT fish
3N	TE-speed (WT, N-S vs S)	8 WT fish	8 WT fish
3O	ΔTE-speed (WT, N-S vs S)	4 WT-WT pairs	4 WT-WT pairs
3P	TE-turn (WT, N-S vs S)	8 WT fish	8 WT fish
4B	Speed (WT vs TG, S)	8 WT fish	4 TG fish
4C	Turning speed (WT vs TG, S)	8 WT fish	4 TG fish
4D	Center Occupancy (WT vs TG, S)	8 WT fish	4 TG fish
4E	Mutual Distance (WT-WT vs WT-TG, S)	4 WT-WT pairs	4 WT-TG pairs
4F	Attraction (WT-WT vs WT-TG, S)	4 WT-WT pairs	4 WT-TG pairs
4G	Near rate (WT-WT vs WT-TG, S)	4 WT-WT pairs	4 WT-TG pairs
4H	Attack posture rate (WT vs TG, S)	8 WT fish	4 TG fish
4I	TE-merged (WT vs TG, S)	4 WT-WT pairs	4 WT-TG pairs
4J	TE-speed (WT vs TG, S)	4 WT-WT pairs	4 WT-TG pairs
4K	TE-turn (WT vs TG, S)	4 WT-WT pairs	4 WT-TG pairs
5B	FBVP (WT vs TG, N-S)	8 WT fish	4 TG fish
5C	FBVP (WT vs TG, S)	8 WT fish	4 TG fish
5E	LEP (WT vs TG, N-S)	8 WT fish	4 TG fish
5F	LEP (WT vs TG, S)	8 WT fish	4 TG fish
5H	REP (WT vs TG, N-S)	8 WT fish	4 TG fish
5I	REP (WT vs TG, S)	8 WT fish	4 TG fish
6E	Distance < 20 (WT vs TG, N-S)	8 WT fish	4 TG fish
6F	Distance > 100 (WT vs TG, N-S)	8 WT fish	4 TG fish
6G	Distance < 20 (WT vs TG, S)	8 WT fish	4 TG fish

6H	Distance > 100 (WT vs TG, S)	8 WT fish	4 TG fish
7A	LEP vs REP (TG, N-S)	4 TG fish	4 TG fish
7B	LEP vs FBVP (TG, N-S)	4 TG fish	4 TG fish
7C	LEP vs REP (TG, S)	4 TG fish	4 TG fish
7D	LEP vs FBVP (TG, S)	4 TG fish	4 TG fish
7F(left)	LEI (WT vs TG, N-S)	8 WT fish	4 TG fish
7F (right)	LEI (WT vs TG, S)	8 WT fish	4 TG fish

Table S2: Trial- wise number of bites performed by fish in the experiments.

Pair	Trial	Fish 1 bite	Fish 2 bite
1	1	1	2
1	2	2	12
1	3	16	0
1	4	8	3
1	5	6	1
1	6	18	13
2	1	10	30
2	2	5	3
2	3	4	4
2	4	3	5
2	5	4	2
2	6	11	9
3	1	4	12
3	2	1	38
3	3	1	2
3	4	1	5
3	5	2	21
4	1	9	4
4	2	5	27
4	3	8	81
4	4	3	7
4	5	9	18
4	6	12	44
5	1	14	2
5	2	13	14
5	3	11	4
5	4	17	6
5	5	12	3
5	6	12	16
5	7	7	3
6	1	4	6
6	2	11	11
6	3	2	3

6	4	6	14
6	5	7	7
6	6	4	7
6	7	8	18
7	1	2	4
7	2	0	5
7	3	3	3
7	4	1	5
7	5	3	1
7	6	1	4
7	7	2	0
8	1	2	1
8	2	2	0
8	3	2	0
8	4	9	5
8	5	1	5
8	6	9	4
8	7	12	7

Table S3 **Frame numbers of approaches made by individual fish towards the opponent paired fish in three pairs.**

Video 1 (6BC_20D)			Video 2 (8TC_17D)			Video 3 (2TC_13D)	
WT(6BC)	TG(20D)		WT(17D)	TG(8TC)		WT(13D)	TG(2TC)
148-162	314-360		318-321	1030-1035		67-80	398-410
258-317	396-415		452-458	1226-1230		156-169	1189-1194
456-464	442-456		569-571	1288-1294		354-370	1576-1584
476-490	524-530		773-778	1829-1837		543-554	1661-1667
530-537	819-828		906-925	1859-1865		560-574	2912-2917
569-590	890-900		954-973	2277-2291		710-723	5519-5528
604-614	1277-1294		979-988	2409-2420		730-743	7272-7277
711-721	1526-1546		1006-1012	2751-2765		820-833	8898-8920
813-822	2082-2092		1120-1128	3176-3184		858-866	
827-847	2447-2463		1219-1224	3392-3405		968-977	
1024-1036	2509-2522		1241-1255	3681-3693		1141-1151	
1084-1099	2998-3024		1288-1294	5109-5121		1248-1258	
1128-1141	3683-3701		1424-1435	5530-5534		1368-1379	
1201-1229	5148-5197		1570-1573	5981-5990		1842-1867	
1448-1463	5265-5278		1683-1698	5995-6002		2879-2901	
1568-1584	5668-5680		1718-1731	6170-6178		3412-3425	
1752-1773	5939-5954		1766-1733	6860-6870		3520-3542	
1798-1807	5982-5992		1781-1790	6928-6946		3729-3744	
1861-1898	6324-6338		1995-2002	7568-7578		3999-4021	
1906-1926	6507-6524		2242-2250	7768-7779		4446-4483	
1954-1985	7145-7178		3066-3077	8022-8037		4605-4630	
2055-2077	7297-7325		3693-3703	8142-8159		4651-4664	
2255-2275	8368-8372		3867-3892	8370-8380		5142-5176	
2413-2436	8549-8558		3919-3925			5274-5287	
2541-2569			4339-4352			5295-5305	
2627-2660			4447-4462			5323-5336	
2903-2930			4620-4640			5880-5891	
3069-3100			4757-4767			5910-5918	
3263-3300			5006-5025			5931-5939	
3408-3423			5142-5151			6254-6264	
3703-3720			5309-5336			6861-6875	
4166-4179			5368-5374			6899-6921	
4199-4214			5378-5388			7018-7028	

4463-4495			5571-5577			7055-7066	
4614-4666			5667-5686			7108-7124	
5329-5349			5840-5846			7159-7172	
5398-5423			5943-5947			7173-7184	
5859-5872			6127-6142			7328-7346	
5992-6013			6244-6250			8138-8162	
6093-6115			6259-6266			8163-8170	
6169-6187			6369-6380			8346-8364	
6293-6317			7012-7018			8514-8521	
6413-6432			7274-7301			8790-8802	
6484-6508			7381-7388				
6650-6679			8213-8221				
6916-6945			8346-8360				
7346-7362			8855-8886				
7513-7532			5428-5437				
7657-7662			5461-5470				
7801-7836							
7896-7934							
8061-8090							
8358-8367							
8393-8412							
8468-8478							
8527-8539							
8559-8569							
8588-8608							
8680-8690							
8708-8715							
8916-8941							
